# Advances in the assessment and study of suicide in late-life depression

**DOI:** 10.3389/fpsyt.2025.1610730

**Published:** 2025-07-30

**Authors:** Tianmei Xu, Yanping Mao, Ye Wang, Wenxuan Zhang, Haojun Cao, Enyan Yu

**Affiliations:** ^1^ The Second School of Clinical Medicine, Zhejiang Chinese Medical University, Hangzhou, Zhejiang, China; ^2^ Department of Psychiatry, The Cancer Hospital of the University of Chinese Academy of Sciences (Zhejiang Cancer Hospital), Hangzhou, Zhejiang, China

**Keywords:** older adults, depression, late-life depression, suicide, assessment tools, treatment methods

## Abstract

With the aging global population, the prevalence of late-life depression (LLD) has shown a significant upward trend. The prolonged presence of a depressed mood can lead to feelings of hopelessness and helplessness, thereby enhancing suicidal impulses and behaviors, which seriously affect the physical and mental health of the older population. However, current research on suicide has mainly focused on adolescents and adults, resulting in a lack of data on the identification, assessment, and intervention of suicide risk in LLD. LLD treatments include psychotherapy, medication, physiotherapy, and Internet intervention techniques. First-line antidepressants, in addition to cognitive behavioral therapy and repetitive transcranial magnetic stimulation, have been shown to be effective in patients with LLD. In this study, we review the research progress in the following three aspects: symptomatological characteristics, assessment tools, and treatment methods of suicide in LLD, providing a reference for clinical workers and researchers in clinical practice.

## 1 Introduction

The world is facing rapid aging, with one out of every six people worldwide projected to be over the age of 64 by 2050 (1). As the population ages, 28.4% global older population experience depression (2). LLD is the occurrence of major depressive disorder in adults 60 years of age or older (3). Compared to younger patients with depression, late-life depression (LLD) is characterized by a lack of interest, somatic symptoms, sleep disturbances, and extensive cognitive deficits (3, 4). Social isolation, bereavement, and chronic illnesses are common risk factors for LLD (5). The symptoms of LLD are frequently masked by physical illness and cognitive decline, resulting in lower rates of diagnosis and treatment (6). Negative perceptions and behaviors are highly prevalent risk symptoms for LLD, and patients with LLD are more suicidal than individuals in other age groups (7). They are also more likely to use highly lethal methods, and suicide rates are higher among older men, with >50% of older adults who die by suicide meeting the criteria for major depression (8), which is far more than any other mental illness. Although a large body of research has been conducted on suicide among adolescents and adults, relatively little international research has been conducted on suicide among LLD. In this study, we review the symptomatological characteristics, assessment tools, and treatment advances for suicide in patients with LLD.

## 2 Symptomatological characteristics of suicide in LLD

### 2.1 Comorbidities

Multiple somatic diseases are common in the older population. Islam et al. found that 82% of the older population had at least one chronic disease, and more than 52% had at least two chronic diseases (9). Moreover, multiple somatic diseases have been associated with an increased risk of LLD (10). Among these, cardiovascular disease (CVD), type 2 diabetes, stroke, chronic pain, and cognitive dysfunction are the main diseases that commonly affect suicide in patients with LLD.

CVD is the most common comorbidity in the older population, and depressive symptoms are associated with an elevated risk of CVD (11). Simultaneously, patients with CVD are also more likely to have depressive symptoms (12), which may be related to the significant psychological burden of impaired cardiovascular function and the limitations in activity after the impairment. Studies have shown that 29% of patients with coronary heart disease (CHD) require antidepressant treatment within 1 year (13), which further improves their mental status and reduces the high levels of hopelessness, depression, and low self-esteem associated with CHD.

Type 2 diabetes mellitus (T2DM), another condition that requires long-term management, is associated with a 24% increased risk of developing depression (14), and the relative risk of suicide in patients with T2DM may be significantly increased following an episode of depressive disorder. The results of the meta-analysis further showed that patients with T2DM had higher rates of suicide and mortality than individuals without diabetes, regardless of whether major depressive disorder preceded the onset of T2DM (15), which may be related to the chronic nature of T2DM and long-term use of medication.

Older adults are at higher risk of stroke, and the prevalence of post-stroke depression ranges from 25% to 79% (16, 17). When organic brain lesions caused by stroke involve brain regions related to mood, including the frontal lobe, they can cause imbalances in neurotransmitters such as serotonin and dopamine (18), which can lead to depressive mood. After a stroke, the body initiates an inflammatory response, and the expression of inflammatory cytokines responsible for regulating the neuroendocrine stress system may ultimately inhibit neurotrophic factors in the brain (19), thereby increasing the risk of depression and suicide. Stroke survivors have a 73% higher risk of suicide than non-stroke survivors (20), which may be related to physical dysfunction and a lengthy rehabilitation process.

Chronic pain, such as arthritis and lower back pain, is prevalent in older adults. A retrospective study found that the prevalence of depression among patients with chronic pain in a chronic pain management clinic in South Africa was 32% (21). Increased feelings of pain frequently exacerbate depressive mood and suicidal risk, with approximately one-quarter of patients with chronic pain experiencing suicidal ideation in the past 2 weeks (22), which may be related to prolonged physical discomfort and social isolation. The interaction between various chronic diseases and LLD raises great concerns regarding the increased risk of death.

The older population usually faces cognitive decline, and studies have found that approximately 30% of people aged > 65 years have varying degrees of cognitive dysfunction (23). This not only affects their ability to deal with complex emotions and problems of daily living but also exacerbates their feelings of helplessness and hopelessness, which may accelerate the onset and progression of LLD. Individuals with LLD frequently exhibit cognitive impairments, including impaired executive functioning (24) and decision-making (25) etc. A large cohort study based on people aged >50 years in the United Kingdom with 16 years of follow-up found that linear changes in depressive symptoms were associated with accelerated memory decline. Conversely, memory decline over time was linked to changes in depressive symptoms (26). Notably, existing research confirms that executive function deficits (27) and memory impairment are more pronounced in suicide attempters (28). It follows that early identification of cognitive dysfunction in LLD is key to predicting and preventing suicidal behavior in clinical practice. Patients with LLD with lower cognitive function scores tend to be accompanied by higher suicide risk scores, which highlights the importance of implementing joint screening for cognitive function interventions and suicide risk in individuals with LLD.

### 2.2 Loneliness and social isolation

Socioenvironmental factors influence suicidal ideation in patients with LLD. Loneliness, despair, and depressive symptoms were found to be closely associated with suicide in older people, as demonstrated in a case-control study on suicide among rural Chinese older adults (29). Loneliness is a subjective emotional experience. On a psychological level, older adults can experience various negative emotions, including sadness and despair, due to loneliness. A longitudinal study in the United Kingdom found that after adjusting for all factors, for every 1-point increase in loneliness scores, depressive symptom severity scores increased by an average of 0.16 (30). Persistent loneliness leads to persistent depressive symptoms, which may form a vicious cycle between depression and loneliness, making suicidal thoughts more likely to develop and intensify. At a physiological level, loneliness triggers various alterations in neuroendocrine and immune functions. Chronically lonely older adults tend to have higher levels of stress hormones such as cortisol (31). Chronic elevation of these hormones affects the balance of neurotransmitters in the brain, particularly serotonin and dopamine, which are closely related to mood regulation and can lead to deeper depression and despair in older people, thereby increasing the risk of suicide. Concurrently, long-term social isolation will enhance the activation of the hypothalamic-pituitary-adrenocortical axis, with certain neuroendocrine components potentially reaching the immune organs through the nerve fibers of the sympathetic nervous system. This process may affect immune function, making older people more prone to various diseases (32). The interactions among physical discomfort, psychological loneliness, and depression will make them less tolerant of life, increasing their suicidal tendencies.

The main commonality of social isolation is the lack of social connectedness, particularly in older adults experiencing bereavement, declining physical health and independence, and estrangement from family or friends, which ultimately drives the development of LLD (33) and may lead to impairment of their cognitive functioning. A review showed that most Asian older adults are made to perceive themselves as a burden due to factors such as social isolation and rising healthcare costs (34). This sense of despair can lead them to feel that suicide is the ultimate means of escaping unbearable psychological pain. Evidence suggests a causal relationship between social isolation and suicide; in contrast, adequate social support has a significant protective effect against suicidal behavior (35). Although not all problems should be interpreted as symptoms of depression, early identification and treatment of affective disorders and improved social support remain key interventions for reducing the risk of suicide in older adults (36). Social and mental health problems should be considered synergistic factors to improve the reliability of suicidal ideation assessments.

## 3 Assessment tools for suicide risk in LLD

### 3.1 Common assessment scales

Various assessment tools have been widely used to assess suicide risk in patients with LLD, including examiner-rating and self-rating scales. The following are a few commonly used assessment scales, as shown in **Table 1**:

**Table 1:** Comparative analysis of suicide risk assessment scales in patients with LLD.

Scale	Number of items	Assessment dimensions	Suicide items	Scoring method	Target population and applicable scenarios	Advantages and limitations
GDS	15 or 30	Mood state, interest, somatic symptoms, cognitive functioning	Some items indirectly reflect suicide risk	0–1 point system (Total score ≥ 5 for 15-item or ≥ 11 for 30-item indicates depression)	Older individuals (≥ 60 years) Community screening, preliminary clinical assessment	Tailored for older individuals, minimizes somatic symptom interference, and does not directly assess suicide risk
BDI	21	Cognitive-affective symptoms, somatic symptoms, behavioral and motivational symptoms	Direct suicide items and indirect items	0–3 point system (Total ≥ 14 indicates depression; score ≥ 2 on Item 9 requires attention)	Adolescents to older individuals; Clinical evaluation, research	Widely used; high reliability and validity Limited differentiation of somatic symptoms; limited focus on suicide risk; and lacks geriatric specificity
SSI	19	Intensity, planning, and control of suicidal ideation	All items focus on suicide	0–2 point system (Higher total score indicates greater risk; requires clinical interpretation)	All age groups; suicide risk screening, crisis intervention	In-depth suicide ideation assessment, quantifies risk levels Time-consuming; requires trained professionals
GSIS	31	Suicidal intent, hopelessness, social support, physical health	Directly evaluates suicidal intent	1–5 point system (Total ≥ 85 indicates high risk)	Older individuals (≥ 60 years) Older individual-specific design, integrates social and physical factors	Designed for older individuals, integrating social and somatic factors Limited cross-cultural validation; underutilized
C-SSRS	6	Frequency of suicidal ideation, plans, preparatory behaviors, and past attempts	All items focus on suicide	Categorical risk classification (none/low/moderate/high) based on frequency and intent	All age groups; emergency settings, clinical evaluation	Structured, globally standardized, distinguishes recent vs. lifetime risk. Requires interviewer training
HDRS	17, 21, or 24	Depression severity (includes suicide item)	Direct suicide items and indirect items	0–4 point system (Score ≥ 2 on item 3 requires intervention)	Adults (including older individuals); Clinical diagnosis, treatment monitoring	Gold standard for depression severity, includes suicide assessment requires expertise; and somatic symptoms may confound scoring in older individuals

GDS, geriatric depression scale; BDI, Beck depression inventory; SSI, suicidal ideation inventory; GSIS, geriatric suicidal ideation scale; C-SSRS, Colombian scale for the assessment of suicide severity; HDRS, Hamilton depression rating scale.

The geriatric depression scale (GDS), developed by Yesavage et al. in 1982 (37), is a depression assessment tool designed specifically for older adults with simple, easy-to-understand questions. The GDS-30 has been shown to be applicable to large-scale community-dwelling older Chinese adults with or without mild cognitive impairment (38).

The Beck depression inventory (BDI), developed by Beck et al. in 1961 (39), is a psychological assessment tool widely used to evaluate the degree of depression in individuals. The BDI-II has some modifications to the original scale to better meet the current diagnostic criteria for depression. Studies have applied it to clinical patients with LLD, and the results have shown high reliability (40, 41).

The scale for suicide ideation (SSI) was developed by Beck et al. in 1979 to measure the severity of suicidal ideation for various populations (42). In 1999, Beck et al. published a subscale for measuring suicidal ideation at the worst point in a patient’s life (suicide ideation at its worst point [SSI-W]), which was superior in predicting suicide (43).

The geriatric suicide ideation scale (GSIS), developed by Heisel et al. in 2006 (44), is a mental health assessment tool designed specifically for older adults. The GSIS has strong measurement properties for community-dwelling older adults, with implications for monitoring suicide risks (45).

The Columbia suicide severity rating scale (C-SSRS) is considered the gold standard for clinicians to assess suicide risk. The C-SSRS is highly predictive of suicide risk in older adults by capturing sensitivity regarding past suicidal behaviors and current suicidal intent, which informs some of its use in older adults (46).

The Hamilton depression rating scale (HDRS), published by Hamilton in 1960 (47), assesses patients’ depressive symptoms in multiple dimensions, including suicidal intent and behavior. Several modified versions of HDRS have been proposed. As noted by Carroll (48), the effectiveness of various versions is remarkable.

Based on the comparative analysis of suicide assessment instruments presented in the table, the following conclusions can be drawn regarding their characteristics and clinical applications: Overall, the above charts and comparisons of suicide entries suggest that the SSI shows particular strength in capturing detailed suicidal ideation, the GSIS excels in integrating social determinants, and the C-SSRS is the most immediate in assessing suicide risk, demonstrating behavioral strengths in clinical practice.

In clinical and research settings, these scales can be used in combination according to the specific situation to achieve the best assessment effect. In conclusion, there are few scales developed for the older population in the field of suicide assessment, and further research can be conducted on the suicide risk of older patients with depression and the establishment of norms in the older population to provide a tool for accurate measurement in the future.

### 3.2 Novel suicide risk assessment

Although accurate assessment of suicide risk in patients with LLD is the key to suicide prevention, it currently relies mainly on clinical interviews and scale assessments, which have limitations such as high subjectivity. Objective, quantifiable biomarkers and neuroimaging-based assessment methods and intelligent digital phenotype monitoring have become research hotspots, aiming to provide more accurate risk stratification and intervention targets.

Evidence suggests that elevated levels of inflammatory markers are associated with an increased risk of suicidal behavior in different populations (49). Recent studies have suggested that combinations of biomarkers such as CRP, IL-6, TNF-α and CXCL-2 may have predictive value for suicide risk (50). Of these, IL-6 is thought to be particularly relevant to suicidal behavior in older adults (51). In addition, comorbidity with co-morbid physical illnesses is common in patients with LLD, which significantly increases the inflammatory load and may further superimpose and amplify the risk of suicide. However, there is a relative lack of systematic studies on inflammatory markers in peripheral blood of patients with LLD, and further research is warranted to explore the role of these markers in the prediction of LLD-associated suicidal risk and the identification of intervention targets.

Neural patterns may provide important clinical value for early identification and intervention strategies for suicidal ideation in patients with LLD. The orbitofrontal cortex and dorsolateral prefrontal cortex (DLPFC) are particularly relevant to suicide because of their involvement in decision making, problem solving, and fluency (52). Another recent functional magnetic resonance imaging (fMRI) study found that functional connectivity and directionality of the ventral lateral prefrontal cortex to the caudate nucleus in patients with LLD differentiated between those with no suicidal risk and those with LLD with a history of suicidal ideation or attempts (53). Abnormal activation of the posterior cingulate cortex (PCC), a core node of the Default Mode Network (DMN), contributes to the recurrent negative memory regurgitation in elderly patients (54). In addition, the nucleus accumbens, among others, is a key brain region associated with suicidal behavior in LLD, and we can use machine learning to construct a high-precision prediction tool for suicidal tendency in patients with LLD on the basis of resting-state fMRI data from the relevant brain regions (55).

Ecological momentary assessment (EMA), which allows for the assessment of participants’ behavioral, emotional, and perceptual experiences in both real-time and real-life environments (56), may capture signals of fluctuations in suicidal ideation that cannot be detected by traditional scales. Several studies have piloted ecological transient assessment and digital phenotyping approaches to assess suicidal risk factors in older adults (57, 58). The application of artificial intelligence (AI) to EMA data is an emerging field, with AI technology showing significant potential for analyzing EMA data to identify dynamic patterns and early warning signs related to suicide risk. However, research evidence on the effectiveness of EMA in suicide prevention is scarce, and research in this area faces challenges such as data heterogeneity and lack of algorithmic interpretability (59). Future longitudinal studies should be designed to coordinate data organization and combine the insights of traditional methods with the power of data-driven technology.

Studies have even shown that AI models can achieve high accuracy in suicide risk prediction by analyzing features such as voice frequency and pause patterns, and that voice biomarkers have demonstrated their feasibility for automated screening for suicide risk in real telemedicine scenarios (60). Voice biomarkers extracted from raw speech signals combined with AI show significant potential for the early diagnosis of LLD (61). Gender-related acoustic and rhythmic-related characteristics may serve as valid identifiers of LLD (62). Future studies should explore in depth the mechanisms linking age-specific phonological patterns and suicide risk in LLD to establish more accurate risk stratification models across ages.

Recent advances in suicide risk assessment for late-life depression have increasingly focused on innovative, technology-driven approaches that complement traditional scales. Digital phenotyping, which collects continuous behavioral and physiological data via smartphones and wearable devices, offers real-time, personalized monitoring of mood and risk factors. Machine learning models applied to electronic health records enable the identification of complex risk patterns often missed in clinical interviews. Natural language processing techniques analyze clinical notes to detect subtle indicators of suicidal ideation, while voice biomarker analysis provides a non-invasive method to assess emotional state through speech characteristics. These emerging tools hold promise for improving early detection and intervention; however, challenges, such as digital literacy and cognitive decline in older adults, must be addressed to ensure effective implementation.

## 4 Treatment of suicide in LLD

Although research suggests that older adults’ emotional processing improves with age (63), and emotional stability is maintained through autonomic compensatory mechanisms (64), it is worth noting that age-related structural and functional brain changes specific to patients with LLD may affect emotion regulation in a maladaptive manner; e.g., LLD appears to be associated with a more severe burden of white matter disease with reduced integrity of the frontal-striatal-limbic network, as well as reduced volume and/or cortical thickness in areas such as the PFC, orbitofrontal cortex, anterior and posterior cingulate gyrus, and hippocampus (65), reward processing, and disconnections between regions associated with subjective positive affect (66), all of which may make it impossible to suppress despair-driven impulsive behavior. Because the neurobiological factors underlying depressive disorders and suicidal risk behaviors are intricately linked (67), LLD-focused treatment may mitigate suicide risk through common mechanisms that undermine the drivers of suicide risk at its source. The majority of elderly suicide deaths can be attributed to depression, and Beautrais found that the significantly reduced suicide rates in the elderly following if LLD intervention (68). Therefore, preventing and treating depression can constitute a powerful suicide prevention strategy for this age group.

The treatment of LLD with suicide risk requires a comprehensive intervention that combines pharmacotherapy, psychotherapy, social support, and other multifaceted means. Owing to the complexity of physical conditions and cognitive functions in older patients, treatment plans should be individualized and early intervention should be emphasized. The following are several commonly used therapeutic approaches:

### 4.1 Pharmacotherapy

Medication is currently one of the basic treatments for LLD, and commonly used drugs include antidepressants and antipsychotics. The use of medications helps reduce the suicidal ideation in patients with depression (69), which is essential for LLD. Antidepressants are the most well-studied treatment options, selective 5-hydroxytryptamine reuptake inhibitors are widely recognized as first-line therapeutic agents for LLD (70). Among them, sertraline is a well-tolerated and safe antidepressant suitable for older patients, particularly for those with comorbidities of physical diseases (71). Consensus Statement of the Spanish Psychogeriatric Association states that dual-acting antidepressants are considered more effective than SSRIs for LLD (72). Norepinephrine-5-hydroxytryptamine reuptake inhibitors (SNRIs) are suitable for patients with LLD who have comorbid chronic pain, including duloxetine (73) and venlafaxine (74), which help to alleviate depressed mood and somatic symptoms. Additionally, it should be noted that SNRIs, particularly venlafaxine, may cause an increase in blood pressure in patients with LLD (75), and blood pressure should be monitored when using this class of drugs. Noradrenergic and specific 5-hydroxytryptaminergic antidepressants, such as mirtazapine, are recommended for older patients with anxiety, insomnia, anorexia, and weight loss (76).

Other antidepressants, including tricyclic antidepressants (TCAs) and monoamine oxidase inhibitors (MAOIs), are of limited use in older adults because of their more frequent side effects, particularly on the heart and blood pressure (77, 78). Vortioxetine significantly improves depressive symptoms and cognitive functioning in patients with depression by modulating 5-HT receptors and multimodal action and is usually well tolerated in older adults (79). The National Disease Management Guideline states that in cases of suicidal ideation in the hospitalized setting, nasal administration of eketamine may be considered in addition to antidepressants (80). As a novel antidepressant, esketamine, has been shown to rapidly and powerfully reduce suicidal ideation in patients with major depression (81). Additionally, for refractory LLD, second-generation antipsychotics, including aripiprazole (82) and quetiapine (83), may have potentiating effects. Owing to the presence of multiple comorbidities and susceptibility to drug interactions in older adults, it is important to pay close attention to the adverse effects of drugs when using appropriate medications.

Cognitive dysfunction and depressive symptoms are also closely linked to LLD. Although antidepressant medications may improve cognitive function in patients with LLD, particularly memory and learning, by ameliorating depressive symptoms (84), this effect is relatively limited. In contrast, cognition-promoting medications have a more direct mechanism of action to improve cognition, which, in turn, improves patient compliance with antidepressant treatment and enhances self-efficacy, both of which are important for alleviating depression and preventing suicide. Cholinesterase inhibitors such as donepezil, which are primarily used to improve cognitive function in patients with Alzheimer’s disease, may be considered for the cognitive decline commonly observed in patients with LLD and may have a positive effect on their cognitive function. However, donepezil can have a temporary positive effect on cognitive function when added to antidepressant maintenance therapy for LLD (85).

Another cognition-improving drug, N-methyl-D-aspartate receptor antagonists such as memantine, has been shown to be well tolerated in combination with escitalopram for treating depressive symptoms and cognitive improvement in LLD (86). Additionally, medications should be adjusted according to the physiological changes in older adults to avoid adverse drug reactions. The risk of relapse in patients with LLD is high, and maintenance therapy is necessary to minimize this risk. Hospitalization for monitoring and treatment is necessary for patients at a high risk of suicide.

### 4.2 Psychotherapy

Expert Consensus Guidelines recommend antidepressant medication combined with psychological intervention as the treatment of choice for LLD (70). Psychotherapy is an important component of suicide interventions for patients with LLD, particularly those with mild-to-moderate LLD. Cognitive behavioral therapy (CBT) can help patients identify and adjust to negative thinking patterns, and for those with LLD, it can effectively alleviate depressive symptoms and reduce suicide risk (87). Additionally, certain treatment gaps exist in the older population due to limited mobility, stigma, or geographic barriers, which may be effectively addressed by Internet-based CBT (iCBT). Meta-analysis reinforces the effectiveness of iCBT in alleviating depressive symptoms in older adults (88). iCBT interventions can prevent suicidal-related thoughts and behaviors (89). Virtual Reality-Enhanced Cognitive Behavioral Therapy (VR-CBT) is a new type of psychological intervention that combines traditional Cognitive Behavioral Therapy (CBT) with Virtual Reality (VR) technology. VR-CBT can stimulate multisensory channels, promote emotional regulation and cognitive flexibility in older adults, and compensate for the monotony of traditional talk therapy. The immersive nature of VR-CBT makes it a potential alternative treatment for patients who are intolerant of, or who do not respond well to, traditional antidepressant medications for the treatment of depression and suicidal ideation (90). Mindfulness-Based Cognitive Therapy (MBCT) is a novel treatment for depression that combines mindfulness meditation with elements of CBT to improve rumination in older patients by modulating overactivation of the Default Mode Network (DMN) (91). MBCT may ameliorate cognitive deficits specific to suicide ideators and attempters (92), enabling individuals to regulate negative emotions. MBCT is increasingly being used in the elderly population, with age-related modifications including more sedentary meditation, shorter duration of each session, etc., and age-modified MBCT is more helpful in controlling depressive symptoms in elderly patients (93). Another common psychotherapeutic approach, interpersonal therapy (IPT), can alleviate depressive symptoms by helping patients improve their relationships with others, IPT is an effective treatment option for LLD and may ultimately help reduce the risk of suicide in this high-risk population (94). Problem-solving therapy (PST) is also an effective treatment for LLD (95), and participants with LLD and executive dysfunction who receive PST are more likely to have reduced suicidal ideation (96).

### 4.3 Neuromodulation therapy

Neuromodulation therapy is particularly important for patients with LLD who are receiving antidepressant medications and may encounter adverse reactions, drug interactions, or unresponsiveness to treatment.

The Canadian Network for Mood and Anxiety Treatment (CANMAT) guidelines recommend ECT as the treatment of choice for patients with major depressive episodes who are at high risk for suicide (97). The rate of remission is significantly higher with ECT for severe LLD, and it should be given a more prominent place in managing these patients (98); however, it can lead to cognitive deficits and adverse effects on the cardiovascular system, among others (99).

Modified electroconvulsive therapy (MECT) is a type of ECT performed under general anesthesia, using inotropes and anesthetics to minimize side effects and improve safety. MECT is an effective, well-tolerated, and safe method for treating patients with refractory LLD (100). MECT is preferred for older patients because it reduces discomfort and anxiety during treatment. MECT can be combined with antidepressant medications to improve therapeutic efficacy, particularly during the acute phase of treatment (101).

Repetitive transcranial magnetic stimulation (rTMS) is a noninvasive brain stimulation technique that stimulates specific brain areas using pulsed magnetic fields. The rTMS has been shown to be effective in young adults, and new data support its use in LLD (102), particularly in those who do not respond well to traditional medication. rTMS targeting dorsal and ventral subregions of the lateral prefrontal cortex (LPFC) in LLD reduces depression scores and improves remission rates (103). Efficacy studies have shown that rTMS is effective and well-tolerated in reducing suicidal ideation and depression severity (104). The rTMS is considered a safe treatment with relatively few side effects and potential cognition-enhancing effects (105). Most patients experience mild discomfort (e.g., scalp tingling or mild headache) during treatment; however, these symptoms are usually transient (106). rTMS has fewer side effects and is particularly suitable for older patients compared with medication.

### 4.4 Social support and interventions

A possible explanation for the limited effectiveness of depression-focused interventions in reducing the incidence of suicidal behaviors among older adults is the multifactorial etiology of these behaviors. Although loneliness, limited social support, and financial problems, may drive the association between depression and suicidal behaviors in the elderly (107, 108), there is a lack of clear evidence-based interventions to reduce loneliness in older adults (109). Studies confirm that, social support could help strengthen coping skills to deal with loneliness to prevent and treat LLD (110, 111). Maintaining quality social contact should be recommended as part of depression treatment in addition to medication and other treatments. While there are no studies testing whether improved social connectedness is a mechanism for reducing suicide, we do not yet know what treatments are recommended. Social prescription activities such as soccer, gardening, and art, are accessible and low-cost interventions that not only provide direct health benefits, but also effectively promote social connectedness among older adults by increasing social support and reducing isolation (112, 113), which have been implemented in a variety of settings with promising results currently. In addition, sustained and standardized “warm call” interventions can enhance the sense of social connectedness and autonomy of older persons living alone in the community and counteract their negative perceptions (114). A previous study showed that LLD patients’ experience of being listened to in a trusting relationship can reconstruct their self-esteem and self-help motivation, which provides a clinical rationale for personalized suicide prevention strategies, with the subsequent need to deepen the contextualized validation of crisis interventions in cross-cultural contexts (115).

It is important to note that cultural continuity and community protection should be key factors in suicide prevention (116). For example, in the context of traditional Chinese culture, depression can be alleviated through intergenerational relationships (referring to the patterns of interaction, distribution of responsibilities, and emotional ties between different age groups within a family or social structure) that enhance older people’s self-assessed health and sense of well-being (117), and ultimately prevent suicide. The National Institute for Health and Clinical Excellence similarly emphasized the important role of community services and family support for people with LLD who have suicidal ideation (118).

In addition, the use of some new technologies has been welcomed by older people. PARO for long-term care has been described diversely as a companion, social, and seal robot (119), which has been variously described as companion robot, social robot, and seal robot. It can provide alternative social connections for older adults through multi-sensor interactions that mimic the responses of living organisms, which can have a significant positive impact on reducing depression and loneliness in older adults (120, 121). Technology-based interventions such as PARO can form a buffer for suicide risk protection by alleviating the negative emotions of emotional numbing, social disconnection, and feelings of worthlessness of geriatric depression.

Primary care is a strategic setting for the treatment and prevention of LLD suicide, drawing on the Prevention of Suicide in Primary Care Elderly: Collaborative Trial (PROSPECT) of interdisciplinary interventions, which comprise the collaboration of primary care GPs, psychiatrists, and care managers for the management of depression, with primary care GPs being responsible for day-to-day health management and initial medication, psychiatrists focusing on the development of individualized treatment strategies and supervising the overall process. The core innovative role of the care manager is to coordinate the communication between doctors, patients, and family members through dynamic assessment, adherence management, and standardized treatment, with the aim of achieving standardized downstreaming and efficient collaboration in the provision of mental health services (122). Studies have demonstrated that this intervention leads to a remission of depressive symptoms in 49.7% of the patients with LLD and is effective in reducing the risk of suicide (123).

There are still many gaps in current research. In the future, we need to examine the mechanism of social contact intervention for suicide and its direct effect on suicidal behavior in high-risk populations, and to accumulate contextualized experience in exploring suicidal crisis intervention in different cultural contexts. Future studies need more long-term longitudinal follow-up to explore the trajectory of depression in old age and the effects of different interventions over a longer time horizon.

## 5 Discussion

Suicide risk remains high in patients with LLD. However, a large variability exists in the presentation of LLD and adult depression, and many aspects of its diagnosis, assessment, and treatment remain unknown. Structured rating scales and novel suicide risk assessment enable clinicians and researchers to more precisely identify and quantify suicidality, thereby facilitating early intervention. This could be further explored in the future to identify measures suitable for the early screening and assessment of suicide risk in patients with LLD. Through interdisciplinary collaborative innovation, a paradigm shift from reactive management to proactive prevention of suicide risk in LLD may be achievable. Current therapeutic approaches encompass psychotherapy, pharmacotherapy, neuromodulation therapy, and internet-based intervention technologies. Emerging evidence suggests that antidepressant medications and non-pharmacological adjunctive therapies, such as transcranial magnetic stimulation, can efficiently mitigate suicide risk among patients with LLD. Notably, the generalizability of assessment tools and interventions for suicide risk in patients with LLD may have limitations due to cultural and geographic biases, such as conflicting values, differences in healthcare infrastructure, etc., and thus culturally adapted assessment tools should be developed in the future.
